# 2-Chloro-6-fluoro­benzoic acid

**DOI:** 10.1107/S1600536811016734

**Published:** 2011-05-07

**Authors:** Richard Betz, Thomas Gerber

**Affiliations:** a Nelson Mandela Metropolitan University, Summerstrand Campus, Department of Chemistry, University Way, Summerstrand, PO Box 77000, Port Elizabeth 6031, South Africa

## Abstract

The title compound, C_7_H_4_ClFO_2_, is a twofold halogenated derivative of benzoic acid. The C—C—C angles within the aromatic moiety cover a range 116.11 (14)–123.96 (15)°, with the maximum and the minimum value next to each other. In the crystal, O—H⋯O hydrogen bonds form carb­oxy­lic acid dimers, which are further connected by C—H⋯F contacts into undulating sheets perpendicular to the *a* axis.

## Related literature

For the crystal structure of benzoic acid (applying neutron radiation), see: Wilson *et al.* (1996 ▶). For the crystal structure of *ortho*-fluoro­benzoic acid, see: Krausse & Dunken (1966 ▶) and of *ortho*-chloro­benzoic acid, see: Ferguson & Sim (1961 ▶); Polito *et al.* (2008 ▶). For graph-set analysis of hydrogen bonds, see: Etter *et al.* (1990 ▶); Bernstein *et al.* (1995 ▶).
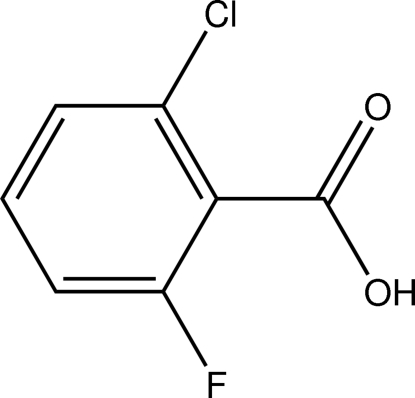


## Experimental

### 

#### Crystal data

C_7_H_4_ClFO_2_
*M* *_r_* = 174.55
Monoclinic, 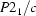

*a* = 3.7655 (2) Å
*b* = 13.9660 (7) Å
*c* = 13.2300 (7) Å
β = 98.034 (3)°
*V* = 688.92 (6) Å^3^
*Z* = 4
Mo *K*α radiation
μ = 0.51 mm^−1^
*T* = 200 K
0.51 × 0.19 × 0.15 mm

#### Data collection

Bruker APEXII CCD diffractometer
11312 measured reflections
1671 independent reflections
1267 reflections with *I* > 2σ(*I*)
*R*_int_ = 0.081

#### Refinement

*R*[*F* ^2^ > 2σ(*F* ^2^)] = 0.032
*wR*(*F* ^2^) = 0.081
*S* = 1.02
1671 reflections
101 parameters
H-atom parameters constrained
Δρ_max_ = 0.23 e Å^−3^
Δρ_min_ = −0.21 e Å^−3^

### 

Data collection: *APEX2* (Bruker, 2010 ▶); cell refinement: *SAINT* (Bruker, 2010 ▶); data reduction: *SAINT*; program(s) used to solve structure: *SHELXS97* (Sheldrick, 2008 ▶); program(s) used to refine structure: *SHELXL97* (Sheldrick, 2008 ▶); molecular graphics: *ORTEP-3* (Farrugia, 1997 ▶) and *Mercury* (Macrae *et al.*, 2006 ▶); software used to prepare material for publication: *SHELXL97* and *PLATON* (Spek, 2009 ▶).

## Supplementary Material

Crystal structure: contains datablocks I, global. DOI: 10.1107/S1600536811016734/gw2099sup1.cif

Structure factors: contains datablocks I. DOI: 10.1107/S1600536811016734/gw2099Isup2.hkl

Supplementary material file. DOI: 10.1107/S1600536811016734/gw2099Isup3.cml

Additional supplementary materials:  crystallographic information; 3D view; checkCIF report

## Figures and Tables

**Table 1:** Hydrogen-bond geometry (Å, °)

*D*—H⋯*A*	*D*—H	H⋯*A*	*D*⋯*A*	*D*—H⋯*A*
O1—H1⋯O2^i^	0.84	1.81	2.6436 (17)	172
C5—H5⋯F1^ii^	0.95	2.46	3.175 (2)	132

Symmetry codes: (i) 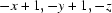; (ii) 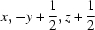.

**Fig. 1.**
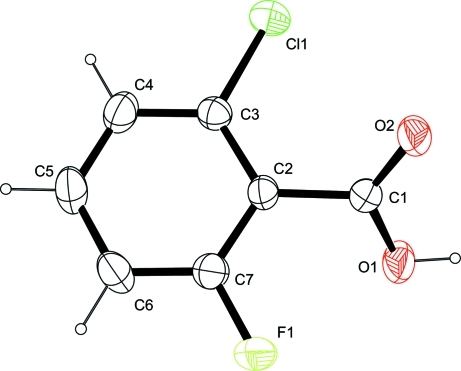
The molecular structure of the title compound, with atom labels and anisotropic displacement ellipsoids (drawn at 50% probability level).

**Fig. 2.**
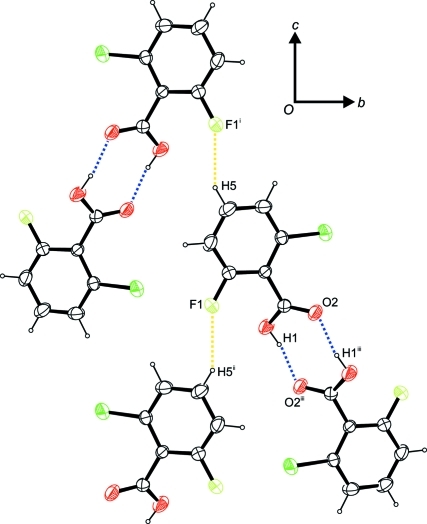
Hydrogen bonds (blue dashed lines) and intermolecular C–F···H contacts (yellow dashed lines), viewed along [-1 0 0]. Symmetry operators: ix, -y + 1/2, z + 1/2; iix, -y + 1/2, z - 1/2; iii -x + 1, -y + 1, -z.

**Fig. 3.**
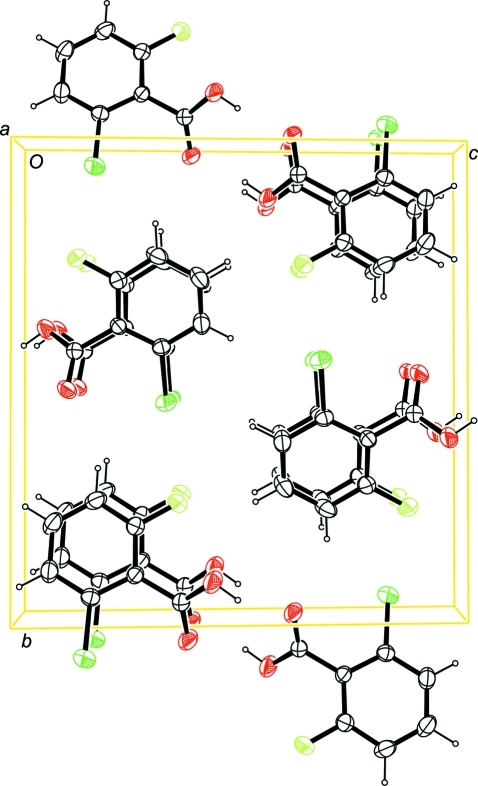
Molecular packing of the title compound, viewed along [-1 0 0] (anisotropic displacement ellipsoids drawn at 50% probability level).

## supplementary crystallographic information

### Comment

Benzoic acid has found widespread use as a ligand in coordination chemistry for a variety of transition metals and elements from the s- and p-block of the periodic system of the elements. It can act as a neutral or – upon deprotonation – an anionic ligand and serve as mono- or bidentate ligand. By varying the substituents on the phenyl moiety, the acidity of the carboxylic acid group can be fine-tuned. Particular interest rests in benzoic acid derivatives showing an asymmetric pattern of substituents on the aromatic moiety due to different possible orientations of the ligand in coordination compounds and the possible formation of stereoisomeric products. At the beginning of a comprehensive study aimed at rationalizing the coordination behaviour of various benzoic acid derivatives towards a number of transition metals in dependence of the *p*H value of the reaction batches it seemed interesting to determine the crystal structure of the title compound to enable comparative studies.

C–C–C angles within the phenyl ring span a range of 116.11 (14) ° to 123.96 (15) ° with the smallest angle found on the C-atom bearing the carboxylic acid group. The biggest angle is found on the fluorine-bonded C-atom and thus directly adjacent to the smallest one (Fig. 1).

Possibly due to steric factors, the carboxylic acid group is not in plane with the phenyl ring. The least-squares plane defined by its C-atom and O-atoms encloses an angle of 47.83 (6) ° with the least-squares plane defined by the C-atoms of the carbocycle and the halogen-atoms.

In the crystal structure hydrogen bonds between the OH-group and the carbonylic O-atom of the carboxylic acid group give rise to the formation of dimeric units. These units are further connected by C–F···H contacts (whose ranges fall by more than 0.2 Å below the sum of van-der-Waals radii of the respective atoms) to wave-like sheets perpendicular to the crystallographic *a* axis. The hydrogen atom involved in the latter contacts is present in *para*-position to the carboxylic acid group on the aromatic carbocycle (Fig. 2). In terms of graph-set analysis, the descriptor for the hydrogen bonds on the unitary level is *R*^2^_2_(8) while the C–F···H contacts necessitate a *C*^1^_1_(5) descriptor on the same level. No π-stacking is observed in the crystal structure.

The packing of the title compound is shown in Figure 3.

### Experimental

The compound was obtained commercially (fluorochem). Crystals suitable for X-ray diffraction were obtained upon slow cooling of a hot aqueous solution of the compound.

### Refinement

Carbon-bound H-atoms were placed in calculated positions (C—H 0.95 Å) and were included in the refinement in the riding model approximation, with *U*(H) set to 1.2*U*_eq_(C). The H atom of the carboxylic acid group was allowed to rotate with a fixed angle around the C—O bond to best fit the experimental electron density (HFIX 147 in the *SHELX* program suite (Sheldrick, 2008)).

### Crystal data

**Table:**

C_7_H_4_ClFO_2_	*F*(000) = 352
*M**_r_* = 174.55	*D*_x_ = 1.683 Mg m^−^^3^
Monoclinic, *P*2_1_/*c*	Mo *K*α radiation, λ = 0.71073 Å
Hall symbol: -P 2ybc	Cell parameters from 4834 reflections
*a* = 3.7655 (2) Å	θ = 2.9–27.9°
*b* = 13.9660 (7) Å	µ = 0.51 mm^−^^1^
*c* = 13.2300 (7) Å	*T* = 200 K
β = 98.034 (3)°	Needle, colourless
*V* = 688.92 (6) Å^3^	0.51 × 0.19 × 0.15 mm
*Z* = 4	

### Data collection

**Table:**

Bruker APEXII CCD diffractometer	1267 reflections with *I* > 2σ(*I*)
Radiation source: fine-focus sealed tube	*R*_int_ = 0.081
graphite	θ_max_ = 28.3°, θ_min_ = 3.1°
φ and ω scans	*h* = −4→4
11312 measured reflections	*k* = −18→18
1671 independent reflections	*l* = −17→17

### Refinement

**Table:**

Refinement on *F*^2^	Primary atom site location: structure-invariant direct methods
Least-squares matrix: full	Secondary atom site location: difference Fourier map
*R*[*F*^2^ > 2σ(*F*^2^)] = 0.032	Hydrogen site location: inferred from neighbouring sites
*wR*(*F*^2^) = 0.081	H-atom parameters constrained
*S* = 1.02	*w* = 1/[σ^2^(*F*_o_^2^) + (0.0326*P*)^2^ + 0.237*P*] where *P* = (*F*_o_^2^ + 2*F*_c_^2^)/3
1671 reflections	(Δ/σ)_max_ = 0.001
101 parameters	Δρ_max_ = 0.23 e Å^−^^3^
0 restraints	Δρ_min_ = −0.21 e Å^−^^3^

### Fractional atomic coordinates and isotropic or equivalent isotropic displacement parameters (Å^2^)

**Table:**

	*x*	*y*	*z*	*U*_iso_*/*U*_eq_	
Cl1	0.56510 (12)	0.54455 (3)	0.33109 (3)	0.03809 (14)	
F1	0.8294 (3)	0.24457 (7)	0.13846 (8)	0.0484 (3)	
O1	0.4025 (4)	0.39103 (9)	0.06067 (9)	0.0441 (3)	
H1	0.3547	0.4253	0.0084	0.066*	
O2	0.7380 (4)	0.51658 (9)	0.11476 (9)	0.0418 (3)	
C1	0.6158 (4)	0.43686 (11)	0.12879 (12)	0.0289 (3)	
C2	0.7161 (4)	0.38510 (11)	0.22715 (11)	0.0254 (3)	
C3	0.7186 (4)	0.42864 (11)	0.32219 (12)	0.0266 (3)	
C4	0.8310 (5)	0.37956 (13)	0.41170 (13)	0.0359 (4)	
H4	0.8276	0.4100	0.4758	0.043*	
C5	0.9477 (5)	0.28638 (14)	0.40775 (13)	0.0391 (4)	
H5	1.0304	0.2534	0.4694	0.047*	
C6	0.9461 (5)	0.24039 (13)	0.31564 (14)	0.0362 (4)	
H6	1.0253	0.1760	0.3127	0.043*	
C7	0.8270 (4)	0.29017 (12)	0.22850 (12)	0.0311 (4)	

### Atomic displacement parameters (Å^2^)

**Table:**

	*U*^11^	*U*^22^	*U*^33^	*U*^12^	*U*^13^	*U*^23^
Cl1	0.0461 (3)	0.0291 (2)	0.0393 (2)	0.00636 (18)	0.00668 (17)	−0.00432 (17)
F1	0.0872 (9)	0.0278 (5)	0.0328 (6)	0.0057 (5)	0.0178 (5)	−0.0044 (4)
O1	0.0638 (9)	0.0349 (7)	0.0284 (6)	−0.0170 (6)	−0.0120 (6)	0.0065 (5)
O2	0.0631 (8)	0.0291 (6)	0.0295 (6)	−0.0149 (6)	−0.0066 (5)	0.0066 (5)
C1	0.0364 (8)	0.0252 (8)	0.0241 (8)	−0.0017 (6)	0.0006 (6)	0.0004 (6)
C2	0.0282 (8)	0.0244 (7)	0.0231 (7)	−0.0018 (6)	0.0021 (6)	0.0028 (6)
C3	0.0255 (7)	0.0256 (8)	0.0285 (8)	0.0010 (6)	0.0036 (6)	−0.0006 (6)
C4	0.0411 (9)	0.0422 (10)	0.0239 (8)	0.0015 (8)	0.0033 (7)	0.0017 (7)
C5	0.0442 (10)	0.0412 (10)	0.0315 (9)	0.0074 (8)	0.0039 (7)	0.0133 (7)
C6	0.0417 (9)	0.0274 (9)	0.0407 (10)	0.0078 (7)	0.0096 (7)	0.0093 (7)
C7	0.0399 (9)	0.0272 (8)	0.0274 (8)	−0.0010 (7)	0.0093 (7)	0.0007 (6)

### Geometric parameters (Å, °)

**Table:**

Cl1—C3	1.7285 (16)	C3—C4	1.383 (2)
F1—C7	1.3519 (18)	C4—C5	1.377 (3)
O1—C1	1.2902 (19)	C4—H4	0.9500
O1—H1	0.8400	C5—C6	1.377 (3)
O2—C1	1.2287 (19)	C5—H5	0.9500
C1—C2	1.490 (2)	C6—C7	1.367 (2)
C2—C7	1.389 (2)	C6—H6	0.9500
C2—C3	1.395 (2)		
			
C1—O1—H1	109.5	C5—C4—H4	120.1
O2—C1—O1	123.63 (14)	C3—C4—H4	120.1
O2—C1—C2	121.11 (14)	C4—C5—C6	120.84 (16)
O1—C1—C2	115.24 (13)	C4—C5—H5	119.6
C7—C2—C3	116.11 (14)	C6—C5—H5	119.6
C7—C2—C1	120.86 (13)	C7—C6—C5	118.00 (16)
C3—C2—C1	122.98 (14)	C7—C6—H6	121.0
C4—C3—C2	121.23 (15)	C5—C6—H6	121.0
C4—C3—Cl1	118.11 (12)	F1—C7—C6	117.50 (15)
C2—C3—Cl1	120.62 (12)	F1—C7—C2	118.51 (14)
C5—C4—C3	119.81 (16)	C6—C7—C2	123.96 (15)
			
O2—C1—C2—C7	−131.11 (17)	Cl1—C3—C4—C5	178.59 (14)
O1—C1—C2—C7	47.3 (2)	C3—C4—C5—C6	−1.6 (3)
O2—C1—C2—C3	46.4 (2)	C4—C5—C6—C7	0.3 (3)
O1—C1—C2—C3	−135.22 (17)	C5—C6—C7—F1	179.46 (15)
C7—C2—C3—C4	0.9 (2)	C5—C6—C7—C2	1.7 (3)
C1—C2—C3—C4	−176.64 (15)	C3—C2—C7—F1	179.95 (14)
C7—C2—C3—Cl1	−176.68 (12)	C1—C2—C7—F1	−2.4 (2)
C1—C2—C3—Cl1	5.7 (2)	C3—C2—C7—C6	−2.3 (2)
C2—C3—C4—C5	0.9 (3)	C1—C2—C7—C6	175.35 (16)

### Hydrogen-bond geometry (Å, °)

**Table:**

*D*—H···*A*	*D*—H	H···*A*	*D*···*A*	*D*—H···*A*
O1—H1···O2^i^	0.84	1.81	2.6436 (17)	172
C5—H5···F1^ii^	0.95	2.46	3.175 (2)	132

Symmetry codes: (i) −*x*+1, −*y*+1, −*z*; (ii) *x*, −*y*+1/2, *z*+1/2.
